# Chinese capillary malformation-arteriovenous malformation: clinical and genetic analysis of eight cases

**DOI:** 10.3389/fmed.2026.1774495

**Published:** 2026-03-27

**Authors:** Yanyan Lin, Shuyan Dong, Changhua Zhu, Linxin Dong, Lihang Lin, Xuemin Xiao

**Affiliations:** 1 Department of Dermatology, The Union Hospital, Fujian Medical University, Fuzhou, Fujian, China; 2 Department of Dermatology, Fujian Provincial Cancer Hospital, Fuzhou, Fujian, China

**Keywords:** capillary malformation-arteriovenous malformation, *EphB4* gene, novel mutation, *RASA1* gene, vascular malformation

## Abstract

**Background:**

Capillary malformation-arteriovenous malformation (CM-AVM) is an inherited autosomal dominant vascular disorder associated with RAS p21 protein activator (*RASA1*) or EPH receptor B4 (*EPHB4*) mutations. We aimed to investigate the clinical features of eight Chinese families with CM-AVM and the genetic characteristics of *EPHB4* or *RASA1* gene variants.

**Methods:**

The clinical data of eight families with CM-AVM who were admitted to the Department of Dermatology, the Union Affiliated Hospital of Fujian Medical University from January 2022 to September 2023 were collected and analyzed. Whole-Exome Sequencing was used to detect the pathogenic gene in the probands, and Sanger sequencing was used to verify the gene mutation sites in family members. Homology modeling software was used to predict the three-dimensional structure of the protein and analyze the characteristics of gene mutations.

**Results:**

Five *EPHB4* gene variants and two *RASA1* mutations were detected in eight patients. Five of these were novel variations: four *EPHB4* (c.2197G>T, p.E733X; c.2451_2460delGGAGAGGCCG, p.E818Tfs^*^4; c.1899_1900insTTGGCGAGGTGTGTCGGGGGCGG, p.K635Afs^*^23; c.2018A>G, p.H673R) and one *RASA1* (c.1848T>A, p.C616X). There were two missense mutations, two frameshift mutations and one non-sense mutation in *EPHB4* mutation, and two non-sense mutations in *RASA1* mutation.

**Conclusions:**

The clinical characteristics of CM-AVM include multifocal capillary malformations (CMs), telangiectasia, and arteriovenous malformation (AVM) or arteriovenous fistulas (AVF). The novel mutations could expand the spectrum of CM-AVM associated with *EPHB4* or *RASA1* mutations.

## Introduction

Capillary malformations (CMs), or port wine stain (PWS), are congenital vascular malformations that occur in 0.3–0.5% of the population worldwide (1). CMs are multiple small, round or oval, red stains that are randomly distributed on the face, trunk and limbs. A typical clinical feature is a pale central zone in the lesions due to micro shunting (2). In 2003, Eerola et al. (3) presented six families with CM manifestations and found that all were associated with RAS p21 protein activator (*RASA1*) mutations; they named this novel disease as capillary malformation-arteriovenous malformation (CM-AVM, OMIM 608354). The *RASA1* gene encodes the RASA1 protein, also known as p120 Ras GTPase-activating protein (p120 RasGAP). CM-AVM is a rare autosomal dominant disease. Until 2017, Amyere et al. (4) first proposed the arteriovenous malformation (AVM) caused by a loss of Ephrin receptor B4 (EphB4) due to a function mutation and defined it CM-AVM2. According to their report, the incidence of CM-AVM2 due to *EPHB4* mutation was one in 120,000 (4). The clinical characteristics of CM-AVM include multifocal CMs, telangiectasia, and AVM or arteriovenous fistulas (AVF) (3). AVM refers to fast-flow vascular anomalies that are characterized by direct communication between arteries and veins without an intervening normal capillary bed, which occurs in approximately one third of patients with CM-AVM (3). As a result, it can arise in the skin, muscle, bone, and internal organs, causing bleeding, congestive heart failure, or seizures (3). Therefore, it is crucial that the condition is detected at an early stage. However, not all individuals carrying the *RASA1* or *EPHB4* variations will exhibit clinical symptoms. Previous research considered the penetrance of EPHB4-CM-AVM syndrome as 93%, while the penetrance of RASA1-CM-AVM syndrome ranged from 90% to 99% (5). The manifestation of CMs may be related to the “second hit” phenomenon. The scholars proposed that pathogenic variants may develop based on germline or chimeric pathogenic variants, and the accumulation of multiple mutation events ultimately determines the appearance of the CM phenotype (6–9).

In this article, we describe eight CM-AVM patients, two of them are CM-AVM1 patients with one novel *RASA1* gene mutation, five of them are CM-AVM2 patients with four novel *EPHB4* gene mutations and one patient had only clinical manifestations without known gene mutations.

## Materials and methods

### Participants

We collected the clinical data and auxiliary examination data of 8 children with CM-AVM who visited the Department of Dermatology, Fujian Medical University Union Hospital from January 2022 to September 2023. There were 5 males and 3 females, aged from 3 to 6 years, and 7 of them had a family history of CM-AVM. The diagnostic criterion for the diagnosis of CM-AVM syndrome was the presence of 3 or more characteristic CMs (10). Patients with less than 3 lesions were included if they had a positive family history or a confirmed genetic mutation (11). This study was approved by the Ethics Committee of Fujian Medical University Union Hospital, and all guardians of the children signed informed consent.

### Whole-Exome Sequencing (WES) and sanger sequencing

Approximately 2 mL of peripheral blood (EDTA anticoagulant) was collected from the patient and the patient's parents with informed consent. A blood genomic DNA extraction kit (Kangwei century) was used to extract genomic DNA from the sample, and the DanoDrop2000 instrument (Thermo company, USA) was used to inspect the extracted nucleic acids. An initial amount of 3 ug DNA from the child's genome was diluted and processed by ultrasound fragmentation using a Covaris S2 ultrasound instrument (Covaris, USA), with a size of about 150 bp. Genomic libraries were prepared with a standard library construction kit (self-developed by Mackinac), and the libraries were inspected by Nanodrop 2000, and agarose gel electrophoresis. Using GenCap liquid phase capture target gene technology (Beijing MyGenostics Company), 726 genes related to skin diseases were captured. The HiSeq4000 (Illumina) sequencing platform was used for double-ended sequencing. The original sequencing data were removed from contamination and coupling sequences, and the filtered sequences were aligned to the human genome reference sequence (HG19) in the NCBI database using BWA software (http://bio-bwa.sourceforge.net/). All SNPs (single nucleotide polymorphisms) and INDELs (insertions and deletions) were analyzed by the GATK software (v.4.0.0) and annotated by the ANNOVAR software. The quality control data of WES are provided in the Supplementary Table 1. Identification of Clinically Relevant Variants: To identify potential disease-causing mutations associated with the CM-AVM phenotype from the raw data, a three-step filtering strategy was applied: (i) Variants were retained if they had a mutant read depth > 5 and a mut-ratio > 20%; (ii) Variants with a frequency > 5% in public databases (e.g., 1,000 Genomes, ESP6500, gnomAD) were removed; (iii) Synonymous variants that did not affect splicing and were not documented in the HGMD database were excluded. The remaining variants were considered potential disease-causing mutations and subjected to further analysis. The candidate mutation sites obtained by analysis and screening were verified by Sanger sequencing, and the genotype of the parents was determined. The primer sequences are listed in Table 1. Amplification was performed by polymerase chain reaction (PCR) using the following conditions: pre-denaturation at 95°C for 3 min, denaturation at 95°C for 30 s, annealing at 56°C to 63°C for 45 s, extension at 72°C for 45 s, for 41 cycles. After 41 cycles, extension at 72°C for 5 min was performed. PCR products were examined by electrophoresis on a 2% agarose gel. The PCR amplified target fragments were recovered, purified with a PCR purification kit (Qiagen, Germany), and sequenced with an AB13730XL automated sequencer (Applied Biosystems, USA). The sequencing results were compared with the human genome *RASA1* and *EPBH4* gene sequence, and the Phred-PhRAP-Consed software package (version 12.0) was used for analysis. If there were any abnormalities, the reverse sequencing was performed.

**Table 1:** Seven pairs of PCR primers used in this study.

Gene	Number of primer	Forward primer (5^′^ → 3^′^)	Reverse primer (5^′^ → 3^′^)
EPHB4	Primer-1	CTCCCACCTTTCCAACCTG	TGAGCTATGATGGCACCACTG
	Primer-2	CAGCAGTGATGACTCTCTGGG	GAGTTGGAGAGTAGGAGGCTTG
	Primer-3	CCTGGCTTCTTAACCCAAGTG	GACCCTAATGAGGCTGTGAGG
	Primer-4	CCTGGCTTCTTAACCCAAGTG	GACCCTAATGAGGCTGTGAGG
	Primer-5	CAGAGGCCTCGCAACTACATC	TCCCTCTCACTTTATAGGATTCACC
RASA1	Primer-6	TGCAATTCTAGAAATCTGGGGTA	AAAACAGAAAGAAATGCAATATGGT
	Primer-7	TTCTAGGCACTGGGTATTTATAGTCC	TCTTTCACCTTGCTGTATGCC

PCR, Polymerase chain reaction.

### Structure prediction

The amino acid sequences of the EPHB4 obtained from the GeneCards database (https://www.genecards.org/Search/Keyword?queryString=EPHB4). The homology modeling program, I-TASSER software (Iterative Threading ASSEmbly Refinement), was used to predict the structure of wild-type and mutant EPHB4 proteins, respectively. I-TASSER automatically searched for template sequences based on sequence similarity, and then combined homology modeling with ab initio prediction to construct three-dimensional structure of protein. The effect of mutation on the three-dimensional structure of protein was analyzed and visualized by software PyMOL 1.7.

## Results

### Clinical assessment of the patients

**Figure 1:**
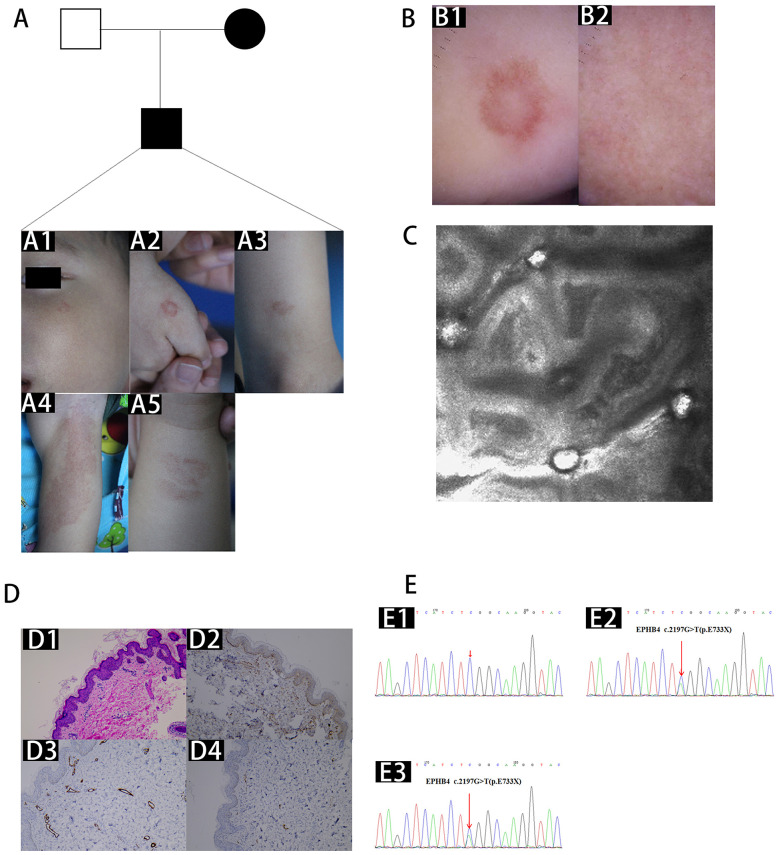
Clinical manifestations, dermoscopy, RCM, pathology, immunohistochemical staining, and Sanger sequencing of the first proband's family. Clinical manifestations. **(A1)** Circular telangiectasia on the left cheek, multifocal erythema with a pale central region on right hand **(A2)** and arm **(A3)**, **(A4, A5)** red patches on the bilateral legs of the first proband. Dermoscopy showed reticular pigmentation with reticular vessels on the dorsal hand **(B1)** and a homogeneous pigmentary background on the face **(B2)**. **(C)** Reflectance confocal microscopy (RCM) showed dilatation and rapid blood flow in the superficial dermis. **(D1)** Skin biopsy revealed increased vessels in the superficial dermis (H and E, 100×). **(D2–D4)** Immunohistochemical staining was positive for vascular endothelial growth factor (VEGF) and negative for smooth muscle actin (SMA) and podoplanin (D2-40) expression (100×). Sanger sequencing of the *EPHB4* mutation of the DNA blood sample of the proband's father **(E1)**, mother **(E2)**, and the proband **(E3)**. Heterozygous c.2197G>T (p.E733X) mutation in exon 13 of *EPHB4* gene.

Case 1 was a 2-year-old boy of Han nationality. Red patches appeared on the child's right hand and arm 6 months after birth. Eventually, both the left cheek and bilateral lower extremities were affected by the stains (Figure 1A). Physical examination showed circular telangiectasia on the left cheek, and multifocal erythema with a pale central region on right hand and arm. Furthermore, fast flow was demonstrated with Doppler interrogation. In addition, there were several large red patches on both legs. The surrounding skin did not differ in temperature. The results of other physical examinations did not reveal any obvious anomalies. It was interesting to note that the erythema and telangiectasia on his mother's right hand and neck resembled that of his rashes. Dermoscopy showed homogeneous pigmentation background with circular vessels in his rashes (Figure 1B). Reflectance confocal microscopy (RCM) presented increased and dilated blood vessels in the dermis with accelerated blood flow of the dorsal hand (Figure 1C). Skin histopathology of the right arm showed that the epidermis had slight hyperplasia, the dermal capillaries were increased, blood vessels were dilated, slender vessels occurred more frequently than round vessels, and lymphocytes infiltration was apparent around the vessels (Figure 1D1). Immunohistochemistry indicated positive vascular endothelial growth factor (VEGF) expression, with negative podoplanin (D2-40) and smooth muscle actin (SMA) expression (Figure 1D2–4). Sanger sequencing was shown in Figure 1E.

**Figure 2:**
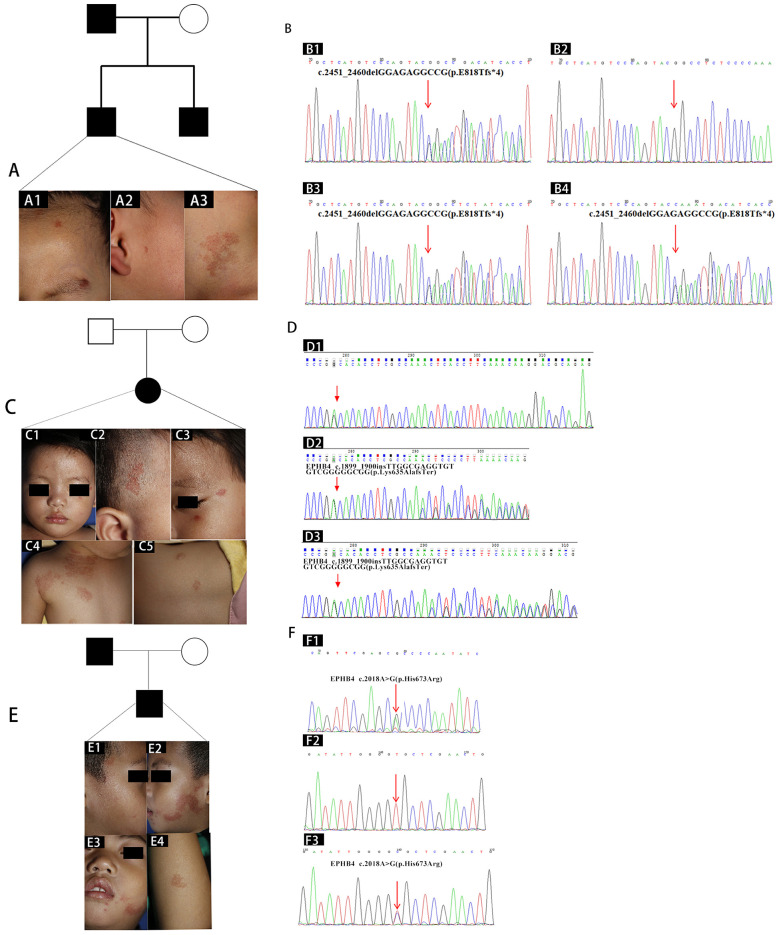
**(A, B)**: Clinical manifestations and Sanger sequencing of the second to the fourth proband's family. **(A)** Clinical manifestations. Coin-size capillary malformation (CMs) on his forehead **(A1)** and right check **(A2)**, with red patches on his neck **(A3)**. **(B)** Sanger sequencing of the *EPHB4* mutation using a DNA blood sample from the proband's father **(B1)**, mother **(B2)**, the proband **(B3)**, and the proband's brother **(B4)**. Heterozygous c.2451_2460delGGAGAGGCCG (p.E818Tfs*4) mutation in exon 14 of *EPHB4* gene. **(C, D)**. Clinical manifestations and Sanger sequencing of the third proband's family. Clinical manifestations. Macules and patches on the left temporal region **(C1)**, scalp **(C2, C3)**, neck **(C4)**, and chest **(C5)**. Sanger sequencing of the *EPHB4* mutation using a DNA blood sample from the proband's father **(D1)**, mother **(D2)**, and the proband **(D3)**. Heterozygous c.1899_1900insTTGGCGAGGTGTGTCGGGGGCGG (p.Lys635AlafsTer) mutation in exon 12 of *EPHB4* gene. **(E, F)**. Clinical manifestations and Sanger sequencing of the forth proband's family. Clinical manifestations. Small red stains and telangiectasia sporadically on his face **(E1–E3)** and right arm area **(E4)**. Sanger sequencing of the *EPHB4* mutation using a DNA blood sample from the proband's father **(F1)**, mother **(F2)**, and the proband **(F3)**. Heterozygous c.2018A>G, (p.His673Arg) mutation in exon 12 of *EPHB4* gene.

Case 2 was a 4-year-old boy who came to our hospital with several coin-size CMs on his forehead and right check (Figure 2A), which developed red patches on his neck 3 months after birth. Physical examination showed several erythema on his forehead and right check, with red patches on his neck. The surrounding skin did not differ in temperature. Sanger sequencing was shown in Figure 2B. The brother presented with one similar erythema on his arm; however, while his father had no clinical symptoms.

Case 3 was a 1-year-old girl who developed rashes one month after birth. Erythema appeared firstly on the left temporal region and gradually spread along the scalp, neck, and chest, showing multiple red macules and patches (Figure 2C). Neither of her parents had clinical manifestations of rash. Sanger sequencing was shown in Figure 2D.

Case 4 was a 4-year old boy who was born with small red stains and telangiectasia sporadically on his face and right arm (Figure 2E). Sanger sequencing was shown in Figure 2F.

Case 5 was a 4-year-old girl who developed red stains sporadically on her face and lip 1 year after birth. The lesions progressively spread to her left face, lip and right arm. Additionally, there were some peripheral blanched halos in her right arm (Supplemental Figure 1A). Interestingly, the father of the proband also had similar rashes distributed on the ear and both upper limbs with the gene mutation at the same site, while the mother had no clinical manifestations. Sanger sequencing was shown in Supplemental Figure 1B.

**Figure 3:**
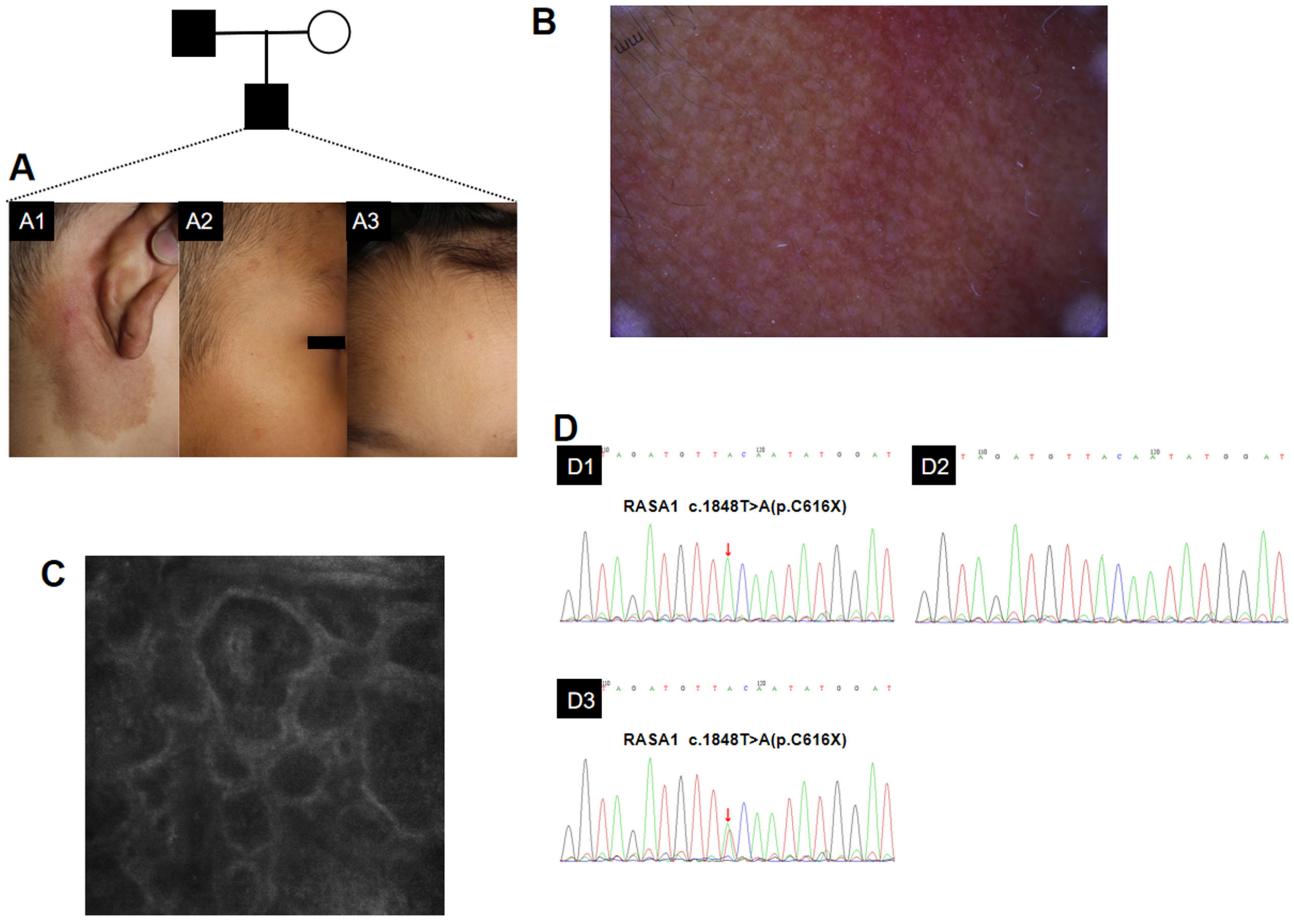
Clinical manifestations, dermoscopy, RCM, and Sanger sequencing of the sixth proband's family. Clinical manifestations. Red stains on his right post aur **(A1)**, face **(A2)**, and forehead **(A3)**. Dermoscopy showed reticular pigmentation with reticular vessels of retroauricular patches. **(B)** RCM presented dilated blood vessels in the dermis of the patches. **(C)** Sanger sequencing of the *RASA1* mutation using a DNA blood sample from the proband's father **(D1)**, mother **(D2)**, and the proband **(D3)**. Heterozygous (c.1848T>A, p.C616X) mutation in exon 14 of *RASA1* gene.

Case 6 was a 6-year old boy, presenting with multifocal spots of erythema on the right post aur, face and forehead (Figure 3A). It's worth noting that the local temperature on palpation of the retroauricular rash was 0.5°C higher than the lateral temperature, which was pulsating. For the family history, the proband's parents had no identical erythema, but his father had the same mutation at this site. Dermoscopy showed reticular pigmentation with reticular vessels of retroauricular patches (Figure 3B), RCM presented dilated blood vessels in the dermis of the patches (Figure 3C). Sanger sequencing was shown in Figure 3D. The patient's family refused the ultrasound (US) and magnetic resonance imaging examinations (MRI).

Case 7 was a 1-year old girl who was born with multiple red macules and patches on the scalp and face. The lesions gradually spread to her breast and legs (Supplemental Figure 2A). As for the family history, the proband's father had no identical erythema and other systemic symptom, however, his mother had the same mutation at this site with scattered dark red patches on left leg. Sanger sequencing was shown in Supplemental Figure 2B.

Case 8 was a 2-year old boy who was born with multiple red macules and patches on the face, arms and legs (Supplemental Figure 3A). Physical examination revealed red patches on the left side of the face and multiple red macules on the face and upper and lower limbs. In addition, the local temperature on palpation of the rash on this patient's face was 0.8°C higher than the lateral temperature, which was pulsating. The proband's mother demonstrated multiple spots of erythema on the face and arm (Supplemental Figure 3B). Interestingly, neither *EPHB4* or *RASA1* mutation was detected in either the proband or his mother. US examination showed that the skin-subcutaneous tissue was slightly thicker than the contralateral side about 0.9 cm (the conntralateral thickness was about 0.7cm), and the blood flow signal was increased compared with the contralateral side, and the turbulent vascular spectrum was measured by doppler ultrasound. MRI demonstrated that the subcutaneous soft tissue around the left orbit and maxillofacial region was slightly thicker than the contralateral side, T2W1 showed high signal, the boundary was unclear, and mild enhancement was seen after contrast enhancement. US and MRI suggested AVM changes with high blood flow. Dermoscopy showed reticular pigmentation with reticular vessels in his red stains of face (Supplemental Figure 3C), RCM demonstrated dilated blood vessels in the dermis of the patches (Supplemental Figure 3D).

All these probands were born at term and born with normal birth weight and development. In addition, there were no further comorbidities, epistaxis histories, or prenatal complications in these probands. Neither the patients nor the relatives had a personal history of epistaxis. Parents were not consanguineous. MRI and angiography of the brain, neck, chest, spine, abdomen, and pelvis revealed no AVM or AVF in these probands (Table 2).

**Table 2:** Clinical and molecular findings in the cases with CM-AVM patients.

Case	Age	Age of onset	Gender	Gene	Nucleotide change	Exon	*M*	Protein change	ACMG pathogenicity classification	*E*	*T*	CMs	Halo	*B*	AVM	Fam Hx
1	2y	6m	Male	EPHB4 (novel)	c.2197G>T	13	Non-sense variant	p.E733X	Likely pathogenic (PVS1+PM2)	No	Face	Face	No	No	No	+
2	4y	3m	Male	EPHB4 (novel)	c.2451_2460delG GAGAGGCCG	14	Frameshift variant	p.E818Tfs^*^4	Likely pathogenic (PVS1+PM2_Supporting)	No	No	Face	No	No	No	+
3	1y	1m	Female	EPHB4 (novel)	c.1899_1900insT TGGCGAGGT GTGTCGGGG CGG	12	Frameshift variant	p.K635Afs^*^23	Likely pathogenic (PVS1+PM2_Supporting)	No	No	Face	No	No	No	-
4	4y	6m	Male	EPHB4 (novel)	c.2018A>G	12	Missense mutation	p.H673R	Uncertain (PM2_Supporting+PP3_ Moderate)	No	Face	Face, arm	No	No	No	+
5	4y	1y	Female	EPHB4	c.175G>A	3	Missense mutation	p.E59K	Uncertain (PM2_Supporting+BP4)	No	Face	Face, lip, neck, arm	Right Arm	No	No	+
6	6y	3m	Male	RASA1 (novel)	c.1848T>A	14	Non-sense variant	p.C616X	Pathogenic (PVS1+PS2+PM2_ Supporting+PP4)	No	No	Face	No	No	Yes	+
7	1y	Birth	Female	RASA1	c.853C>T	4	Non-sense mutation	p.R285X	Pathogenic (PVS1+PS4+PM2_ Supporting)	No	Breast	Breast, limb	No	No	No	+
8	2y	Birth	Male	None	No	No	No	No	No	No	No	Face, limb	No	No	Yes	+

Y, years old; m, month; M, mutation tipe; ACMG, American College of Medical Genetics and Genomics; E, epistaxis; T, telangiectasia; CM, capillary malformation; B, bier spots; AVM, arte riovenous malformation; Fam Hx, family history.

### Disease-associated variants

Five unrelated individuals were identified to have a variant in the *EPHB4* gene, four of the five were novel. Two individuals were identified to have a variant in the *RASA1* gene (Table 2), one was novel. There were two missense mutations, two frameshift mutations and one non-sense mutation in *EPHB4* mutation, and two non-sense mutations in *RASA1* mutation, without obvious distribution hot spot. The gene frequencies of these seven mutation sites were not retrieved in various databases such as Pubmed, HGMD, and ClinVar. Moreover, it has not been reported in other databases (such as the ESP database, the 1,000 Genomes database, and the gnomAD database) for healthy individuals. A total of 100 ethnically matched healthy individuals with no history of vascular malformations, hereditary skin diseases, or severe systemic diseases were enrolled as controls. The healthy controls ranged in age from 0 to 8 years (mean age: 4.07 + 2.20 years), and were gender-matched with the patient group. Targeted variant validation was performed in the entire coding exons and exon-intron boundaries (+20 bp) of *EPHB4* and *RASA1* in all 100 healthy controls using the same exome sequencing pipeline. None of the mutations identified in CM-AVM patients were detected in these healthy controls.

**Figure 4:**
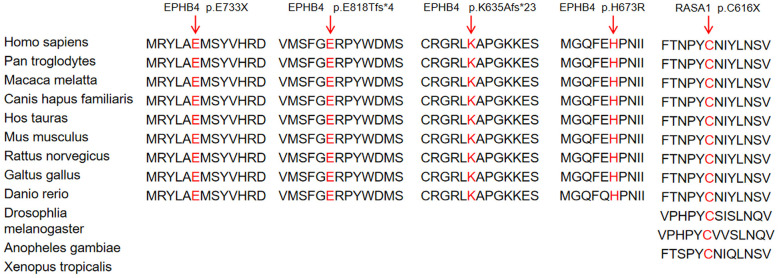
The impacted amino acid residues of EphB4 and *RASA1* protein are highly conserved across species.

Mutations in the *EPHB4* gene were found in case 1–5 (NM_004444:c.2197G>T, p.E733X; c.2451_2460delGGAGAGGCCG, p.E818Tfs^*^4; c.1899_1900insTTGGCGAGGTGTGTCGGGGGCGG, p.K635Afs^*^23; c.2018A>G, p.H673R; c.175G>A, p.E59K, respectively), *EPHB4* (c.175G>A, p.E59K) was reported earlier in previous literature (3). Family history was reported in these five cases. Mutations in *RASA1* were identified in case 6–7 (NM_002890:c.1848T>A, p.C616X; c.853C>T, p.R285X), both of which were inherited from the father or mother, which were confirmed by subsequent Sanger sequencing (Table 2). *RASA1* (c.853C>T, p.R285X) was reported in previous literature (9). Furthermore, the impacted amino acid residues of novel *EPHB4* and *RASA1* are also highly conserved across species (Figure 4), demonstrating their functional significance throughout evolution. The pathogenicity of these variants are detailed in Table 2, according to the criteria established by the American College of Medical Genetics and Genomics (ACMG).

## Discussion

CM-AVM is a rare genodermatosis with cutaneous capillary malformations and a risk of associated fast-flow malformations. In 2013, Orme et al. (10) proposed the following clinical diagnostic criteria for CM-AVM1: detection results of three or more CMs or *RASA1* gene mutation, or the presence and family history of CMs and AVM. Currently, there are no clear diagnostic criteria and treatment guidelines for CM-AVM2 whose phenotypic spectrum is still expanding. The CM-AVM2 phenotype is characterized by a lower risk of AVMs/AVFs, possible Bier spots, telangiectasia, and recurrent rhinorrhea (4, 12). Both CM-AVM1 and CM-AVM2 feature multifocal CMs, AVMs, and Bier spots; however, CM-AVM2 often presents with a greater number of them.

Furthermore, there may be a pallor in the center of the larger CMs in CM-AVM2. Telangiectasia in CM-AVM2 is usually found around the lips and perioral region in comparison to CM-AVM1. CM-AVM2 may present with telangiectasia, a condition that must be distinguished from hereditary hemorrhagic telangiectasia and hereditary benign telangiectasia; the combination of CMs with telangiectasias can be used to distinguish CM-AVM2 from hereditary benign telangiectasia and hereditary hypertension (4). The most fundamental diagnostic feature of CM-AVM2 is a mutation of the *EPHB4* gene. To date, a total of 83 *EPHB4* mutations have been identified in CM-AVM2, which included 39 heterozygous missense mutations, 15 non-sense mutations, 22 frameshift mutations, and 7 splice site mutations (4, 7, 13–16). Our study also found that the hot spot mutations were in exons 6 and 14, accounting for 12% and 15.7% of reported *EPHB4* mutations, respectively. In the previous literature reports (17), *RASA1* gene mutations account for approximately 70 percent of CM-AVM cases, and recently *EPHB4* gene mutations have been identified to be responsible for 25 percent of CM-AVM cases. According to previous researches, compared with Western countries, the mutation sites in China are more “privat.” In the Caucasian population, certain recurrent “hotspot mutations” have been reported. However, in the Chinese population, the currently discovered mutation sites are very scattered, and most are “private mutations” specific to a certain family. This means that it is difficult to find one or two mutation sites that occur frequently in the Chinese population, and genetic testing needs to cover the entire coding region of the gene (4, 7, 13–16). Moreover, the mutation rate of *RASA1* is higher than that of *EPHB4*. However, in our 8 cases, the proportion of *RASA1* mutation and *EPHB4* mutation accounted for 25 percent and 62.5 percent respectively. It's evident that the mutation of *EPHB4* is higher, this could be due to the small sample size issue and further expansion of the sample size is needed for subsequent analysis.

*EPHB4* encodes a transmembrane receptor (EphB4) that is mainly expressed on venous endothelial cells (ECs). Its ligand, EphrinB2, is also a transmembrane protein expressed on arterial ECs. This EphrinB2–EphB4 interaction is essential for vascular remodeling, proper morphogenesis of capillary beds, vasculogenesis, and neuronal development during embryogenesis (18). Through EphrinB2–EphB4 contacts, RASA1 is recruited intracellularly and inhibits the RAS-MEK-ERK1/2 and RAS-AKT-mTOR signaling pathways. A mutation in *EPHB4* activates both pathways, resulting in abnormal endothelial differentiation and the development of CM and AVM (4). As a downstream protein of the PI3K/AKT signal transduction pathway, the phosphorylation of mammalian target of rapamycin (mTOR) plays a critical role in angiogenesis. Through the overactivation of mTORC1, caused by mutations in *RASA1* or *EPHB4*, CMs, and AVMs are formed as well as vascular overgrowth. Hence, RAS and ERK inhibitors, which are downstream signaling effectors of EphB4–RASA1, may be considered as prospective molecular therapies for the treatment of AVM and CM. The role of *RASA1* was demonstrated by Kawasaki et al. (19) to be an EphB4 downstream effector, and the *RASA1* gene mutation in CM-AVM may explain the similar clinical symptoms between CM-AVM2 and CM-AVM1.

Chen et al. (20) conducted an experiment that induced EC-specific disruption of EPHB4 protein in mice, which resulted in the accumulation of collagen IV in the endoplasmic reticulum (ER) of ECs, leading to EC apoptosis and pathological angiogenesis. Moreover, strong evidence has demonstrated that RASA1 and EphB4 function in the same signaling pathway to protect against the development of CM-AVM, independent of physical interaction (20).

**Figure 5:**
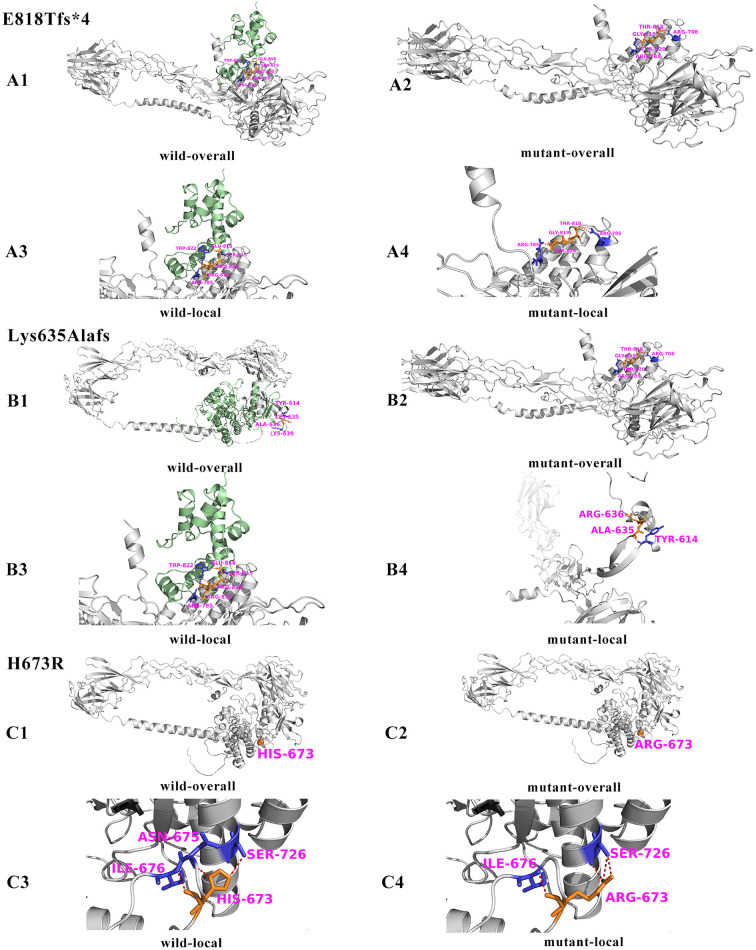
Three-dimensional structure prediction map of the EphB4 protein. **(A)**. Overall **(A1)** and local **(A3)** structure of wild-type, overall **(A2)** and local **(A4)** structure of mutant protein p.E818Tfs*4. **(B)**. Overall **(B1)** and local **(B2)** structure of wild-type, overall **(B3)** and local **(B4)** structure of mutant protein p.K635Afs*23. Overall **(C1)** and local **(C2)** structure of wild-type, overall **(C3)** and local **(C4)** structure of mutant protein p.H673R.

Among the novel *EPHB4* gene mutations, not only two non-sense mutations may affect EPHB4 protein structure and function, but also the other three mutations. E818Tfs^*^4, a frameshift mutation, results in a three-amino acid ERP mutation at positions 818–820 to TGT (yellow sticks structure in Figure 5A). This is followed by a stop codon, resulting in the loss of the amino acid domain at positions 821–987 (green structure in Figure 5A). In the wild-type protein, Glu818 forms hydrogen bond interactions between 815Ser and Trp822, Arg819 and Trp822, and Pro820 and Arg785. In the mutant form, Glu818 is mutated to Thr818, and the mutant Thr818 forms two hydrogen bond interactions with Arg706; however, the hydrogen bond interaction with 815Ser and Trp822 is lost. The Arg819Gly mutation also leads to the destruction of the original hydrogen bond interaction with Trp822 (21). The Lys635Alafs mutation is also a frameshift mutation that results in two amino acids Lys and Ala at positions 635 to 636 being mutated to Ala and Arg (yellow sticks structure in Figure 5B). This is followed by a stop codon, which results in the loss of the amino acid domain at positions 637–987 (green structure in Figure 5B). In the wild type, Lys635 and Tyr614, Ala636, and Lys639 had hydrogen bond interaction. When the frame-shifted mutation occurs, Ala635 and Tyr614 has hydrogen bond interaction, but Arg636 has no hydrogen bond interaction with other amino acids. More importantly, these two frameshift mutations can lead to coding early termination, causing the loss of important C-terminal region of EphB4, According to the Uniprot records (https://www.uniprot.org/uniprotkb/P54760/entry), the important functional region include part of the protein kinase domain (positions 821–899 and 635–899, respectively), sterile alpha motif (SAM) domain (positions 907–971), disordered region (965–987) and PDZ-binding motif (985–987) (22, 23). The loss of these important functional regions may lead to abnormal protein function, which in turn leads to disease transmission. The missense mutation H673R occurs in the loop region of the protein structure (green structure in Figure 5C). In the wild type, His673 forms three hydrogen bonds with the surrounding Asn675, Ile676, and Ser726. When mutated to Arg, the loop structure of the original side chain of His changes to the chain-like structure of Arg, and Arg is a polar amino acid with positive charge. The hydrogen bond interaction between Arg673 and Asn675 disappears, and the hydrogen bond interaction between Arg673 and Ile676 is the same as that of the wild type, but Arg673 forms three hydrogen bonds with Ser726, showing a stronger interaction.

The latest research suggested that reticular pigmentation with reticular vessels was significantly more frequent in CM-AVMs with fast-flow vascular malformations (FFVMs), while an underlying homogeneous pigmentary background was significantly more frequent in CM-AVMs without FFVMs (24). Six of our eight patients had only multiple CMs without high-flow AVMs, of whom six had dermoscopic findings of underlying homogeneous pigmentary background. Two patient with AVM showed reticular pigmentation with reticular vessels, which is broadly consistent with the latest study (24). The difference between CM-AVM and AVM was that the former has a family history, multiple CMs and *RASA1* or *EPHB4* gene mutations. In addition, dermoscopy of CM-AVM presents reticular pigmentation with reticular vessels or a homogeneous pigmentary background. The latter has no family history, CM, and dermoscopy of AVM also shows reticular pigmentation with reticular vessels, but no homogeneous pigmentary background pattern; the most important identification point is the absence of genetic mutations in AVM. Unlike CM-AVM and AVM, the skin lesions of PWS do not show an increase in skin temperature. The three classic vascular modes of dermoscopy are dotted and globular vessels, short clubbed vessels, and curved vessels. Therefore, dermoscopy is a very good auxiliary tool that can help to identify CM-AVM, AVM, and PWS.

In terms of treatment, some scholars had used 595-nm pulsed dye laser (PDL) to treat CM-AVM and showed that CMs responded well to PDL (25, 26). The potentially longer-lasting side effects of PDL include blistering, ulceration, scarring, and hypopigmentation. Therefore, finding an effective treatment with fewer side effects is crucial. Vascular targeted hemoporfin-mediated photodynamic therapy (HMME-PDT) has been shown to be effective and safe for treatment of PWS. Mei et al.'s (27) experimental studies showed that HMME-PDT inhibited the activation of AKT, mTOR, and P70S6 by inhibiting VEGF-A expression, supporting that HMME-PDT blocked the VEGF-A-mediated AKT/mTOR pathway. Thus, HMME-PDT can inactivate blood vessels by inhibiting mTOR. Thus, we predicted that using HMME-PDT to treat CMs could be beneficial.

In the eight cases, the family members had differing clinical manifestations and severity of CM-AVM. Phenotypic inconsistencies within a family may be due to modification factors, epigenetic events, or environmental influences. For multifocal CMs, patients need to undergo genetic testing and appropriate imaging studies to determine AVM, and then precautions, such as regular clinical follow-up and bleomycin injection, should be taken to prevent AVM formation. At the same time, in some children with CM-AVM whose AVM or AVF was not detected at the early stage, if new symptoms appear during follow-up, we believe that it is necessary to improve on the corresponding MRI examination, and recommend genetic testing and genetic counseling for *RASA1* and *EPHB4* mutations in their parents. We still do not fully understand the relationship and mechanism between the phenotype and genotype of *EPHB4* or *RASA1* mutations in CM-AVM. Previous research has likewise failed to establish robust genotype-phenotype correlations. However, Chen et al.'s (7) put forward the distribution of unifocal or multifocal CMs was related to the presence of somatic or germline pathogenic variants (PV), suggesting that clinical progression may be more influenced by age and the main characteristics of CM. Our future studies will focus on sequencing patients who have *EPHB4*-related or *RASA1-*related CM-AVM syndrome to better understand their clinical presentation, and conduct gene function researches to determine the pathogenesis of CM-AVM.

In conclusion, we analyzed the clinical and genetic characteristics of eight pedigrees with CM-AVM. The novel mutations could expand the spectrum of CM-AVM associated with *EPHB4 or RASA1* mutations, and lay a foundation for further study of the genetic diagnosis and genetic counseling of the disease. We suggest establishing a multidisciplinary team (MDT) clinic for CM-AVM patients and their families, so as to provide long-term personalized diagnosis, treatment and follow-up service.

## Data Availability

The raw data supporting the conclusions of this article will be made available by the authors, without undue reservation.
