# Supplementary material for: Chinese capillary malformation-arteriovenous malformation: clinical and genetic analysis of eight cases
Source: Front Med (Lausanne). 2026 Mar 27;13:1774495. doi: 10.3389/fmed.2026.1774495 (PMC13065680; doi:10.3389/fmed.2026.1774495)

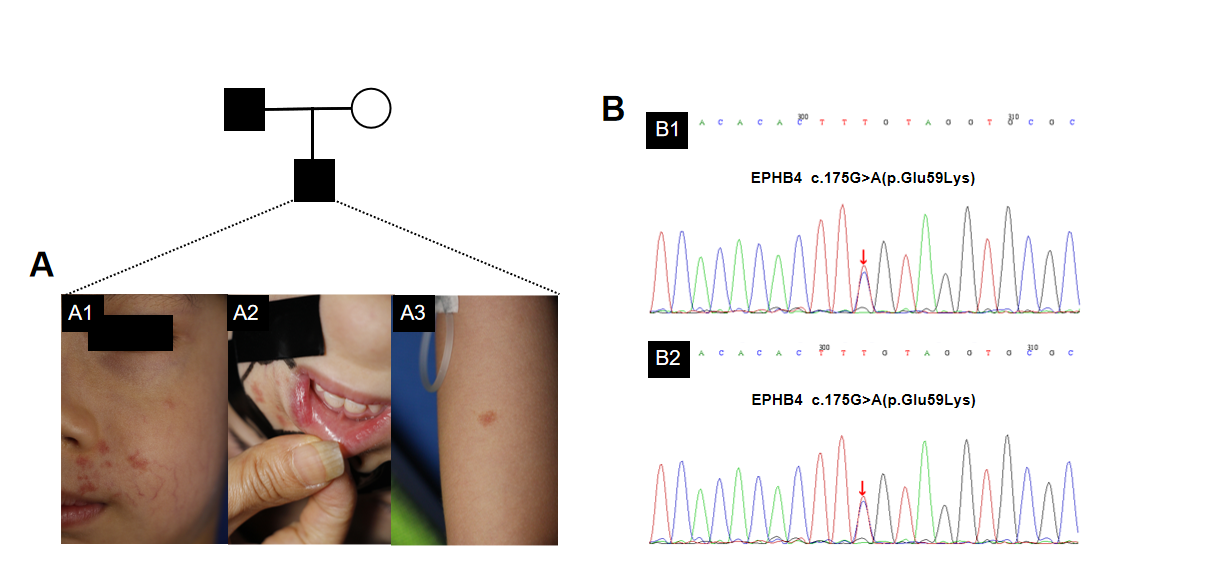


**Supplementary Figure 1**


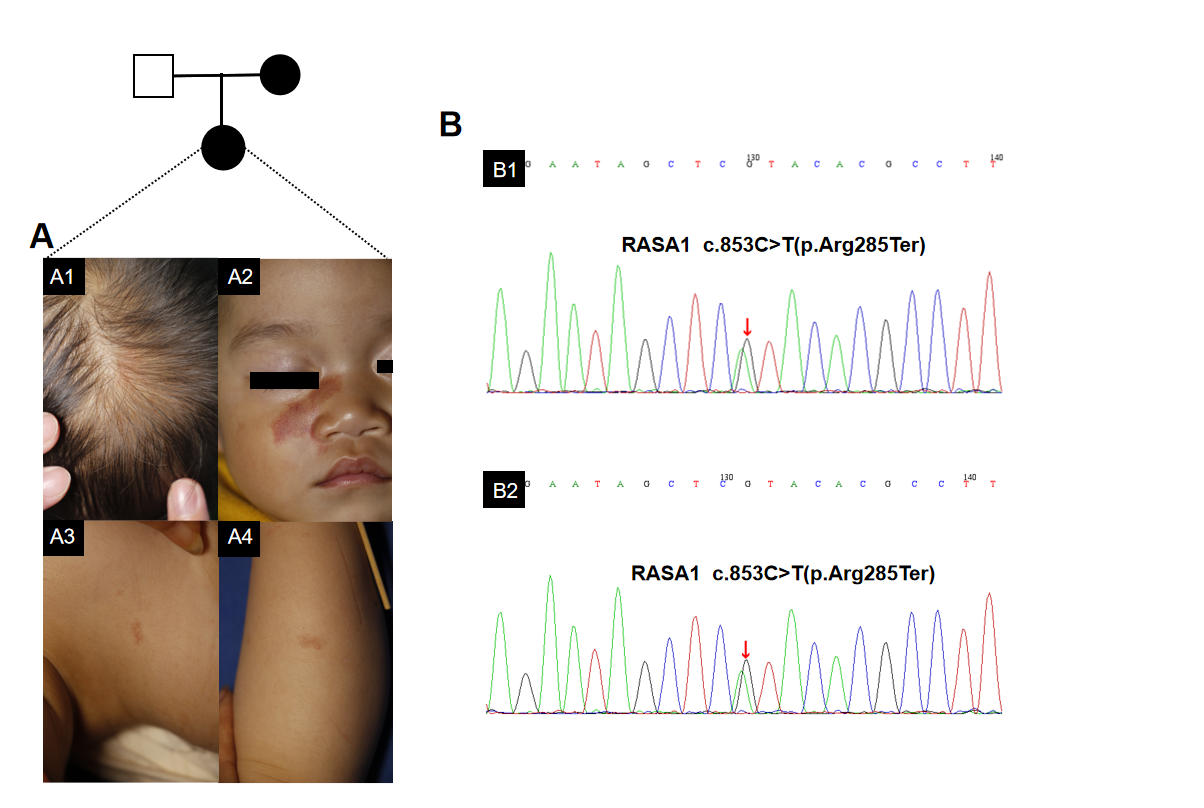


**Supplementary Figure 2**


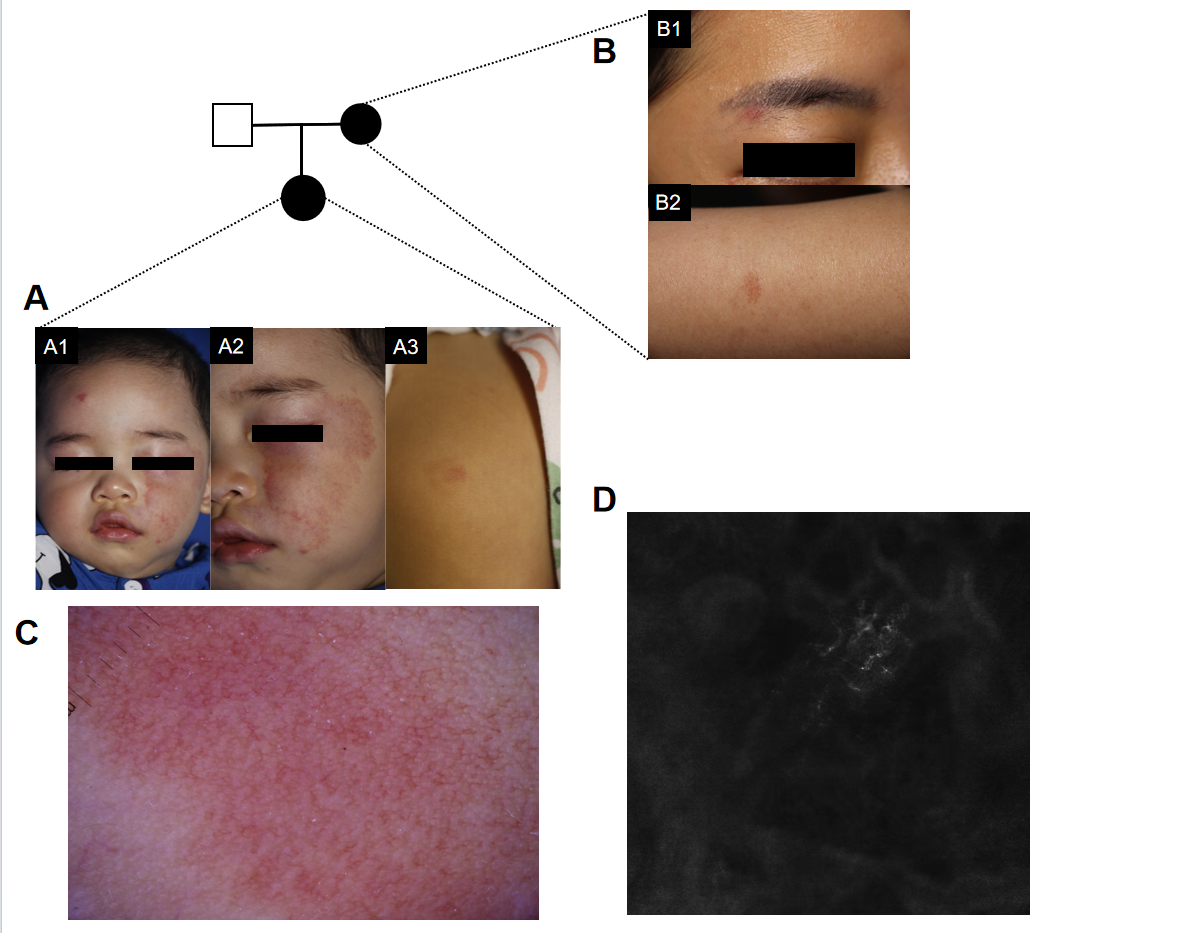


**Supplementary Figure 3**

**Supplemental legend**

**Figure 1.** Clinical manifestations and Sanger sequencing of fifth proband and her father

1. Clinical manifestations. Red stains on her left face (A1), lip (A2) and right arm (A3) .
2. Sanger sequencing of the *EPHB4* mutation using a DNA blood sample from the proband’s father (B1) and the proband (B2). Heterozygous c.175G>A, (p.Glu59Lys) mutation in exon 3 of *EPHB4* gene.

**Figure 2.** Clinical manifestations and Sanger sequencing of seventh proband and her mother

1. Clinical manifestations. Red macules and patches on the scalp (A1), face (A2), breast (A3) and legs (A4).

(B) Sanger sequencing of the *EPHB4* mutation using a DNA blood sample from the proband’s mother (B1) and the proband (B2). Heterozygous c.853C>T, (p.Arg285Ter) mutation in exon 7 of *RASA1* gene.

**Figure 3.** Clinical manifestations of the eighth proband and his mother, dermoscopy and RCM of the proband

1. Clinical manifestations. Red patches and multiple red macules on the left face (A1,A2) and upper and lower limbs (A3).
2. Multiple spots of erythema on the face and arm of her mother.
3. Dermoscopy showed reticular pigmentation with reticular vessels in his red stains of face,
4. RCM demonstrated dilated blood vessels in the dermis of the patches.

**The quality control data of the experiment are as follows:**


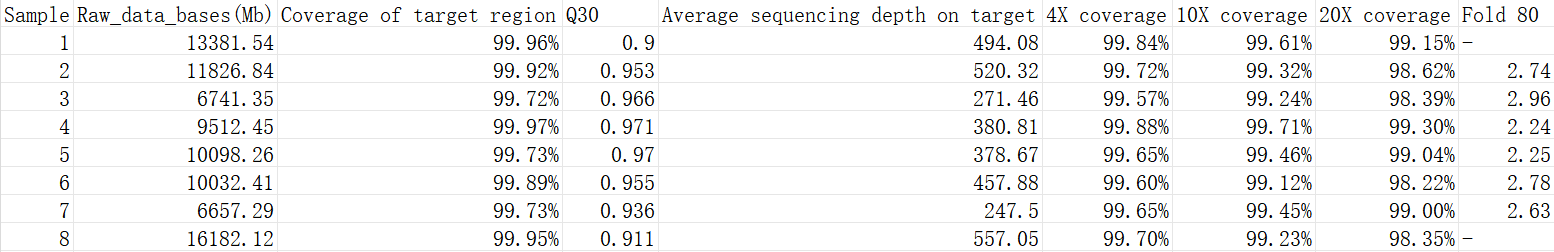

Supplement: Supplementary file 1 [file Supplementary_file_1.doc]
